# 

**Published:** 1911-03-25

**Authors:** 


### THE HOSPITAL

THE MODERN MEDICAL AND INSTITUTIONAL JOURNAL.

Telegraphic Address: Ospedale, London.

Telephone Number: 2734 Gerrard.

NEW SERIES. No. 216, Vol. VIII. No. 1288, Vol. XLIX.

SATURDAY, MARCH 25TH, 1911.

PRICE THREEPENCE

